# 2-(3-Bromo-4-meth­oxy­phen­yl)acetic acid

**DOI:** 10.1107/S1600536810020143

**Published:** 2010-06-05

**Authors:** Ilia A. Guzei, Alan R. Gunderson, Nicholas J. Hill

**Affiliations:** a Department of Chemistry, University of Wisconsin–Madison, 1101 University Ave, Madison, WI 53706, USA

## Abstract

The title compound C_9_H_9_BrO_3_, was synthesized by the regioselective bromination of 4-meth­oxy­phenyl­acetic acid using bromine in acetic acid in a 84% yield. In the mol­ecular structure, the meth­oxy group is almost coplanar with the phenyl ring within 0.06 Å; the acetic acid substituent is tilted by 78.15 (7)° relative to the ring. The C—C—C angles at the OMe, acetyl and Br substituents are 118.2 (2), 118.4 (2) and 121.5 (2)°, respectively, indicating that the Br atom is electron-withdrawing, whereas the other substituents possess electron-donating properties. In the crystal, the mol­ecules form centrosymmetric strongly O—H⋯O hydrogen-bonded dimers of the type *R*_2_^2^(8).

## Related literature

For the use of the title compound in the synthesis of natural products such as *Combretastatin A*-4, see: Zou *et al.* (2008 ▶); for *Verongamine*, see: Wasserman & Wang (1998 ▶) and for model *Vancomycin*-type systems, see: Ghosh *et al.* (2009 ▶). The iodo-analogue featured in the synthesis of (+)-*Phleichrome* and (+)-*Calphostin D*, see: Morgan *et al.* (2010 ▶). For the synthesis of the title compound, see: Coutts *et al.*, (1970 ▶); Morgan *et al.*, (2007 ▶); Zou *et al.* (2008 ▶); Ghosh *et al.* (2009 ▶). For background for our program to introduce natural product synthesis, crystal growing techniques and single crystal X-ray diffraction data analysis into the undergraduate curriculum, see: Findlater *et al.*, (2010 ▶); Guzei *et al.*, (2010*a* ▶). For a discussion of hydrogen-bonding motif assignment, see: Guzei *et al.* (2010*b* ▶). Outlier reflections were omitted based on the statistics test described by Prince & Nicholson (1983 ▶) and Rollett (1988 ▶), and implemented in *FCF_filter* (Guzei, 2007 ▶).
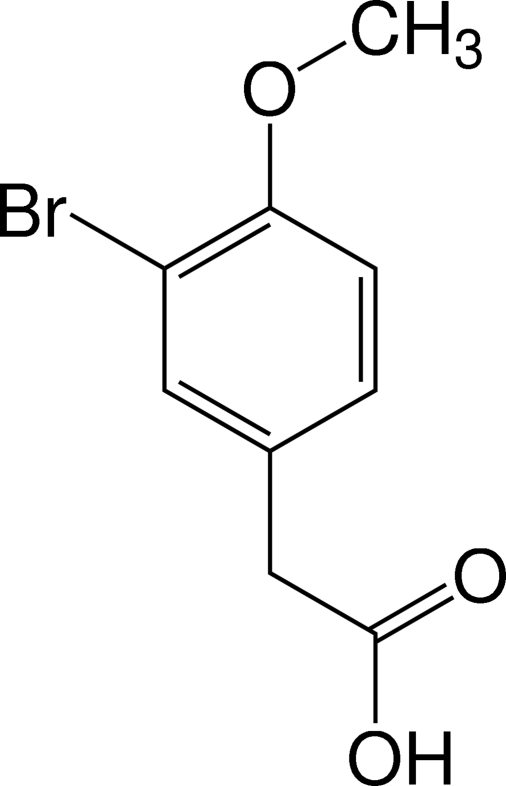


## Experimental

### 

#### Crystal data

C_9_H_9_BrO_3_
*M* *_r_* = 245.06
Monoclinic, 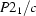

*a* = 12.5022 (4) Å
*b* = 8.2690 (2) Å
*c* = 9.0199 (3) Å
β = 93.573 (1)°
*V* = 930.67 (5) Å^3^
*Z* = 4
Cu *K*α radiation
μ = 5.81 mm^−1^
*T* = 120 K
0.46 × 0.37 × 0.19 mm

#### Data collection

Bruker SMART APEXII area-detector diffractometer
Absorption correction: analytical (*SADABS*; Bruker, 2007 ▶) *T*_min_ = 0.177, *T*_max_ = 0.398
12598 measured reflections
1725 independent reflections
1708 reflections with *I* > 2σ(*I*)
*R*_int_ = 0.030

#### Refinement

*R*[*F* ^2^ > 2σ(*F* ^2^)] = 0.026
*wR*(*F* ^2^) = 0.073
*S* = 1.08
1725 reflections
120 parameters
H-atom parameters constrained
Δρ_max_ = 0.86 e Å^−3^
Δρ_min_ = −0.47 e Å^−3^

### 

Data collection: *APEX2* (Bruker, 2007 ▶); cell refinement: *SAINT-Plus* (Bruker, 2007 ▶); data reduction: *SAINT-Plus*; program(s) used to solve structure: *SHELXTL* (Sheldrick, 2008 ▶); program(s) used to refine structure: *SHELXTL*; molecular graphics: *SHELXTL*; software used to prepare material for publication: *publCIF* (Westrip, 2010 ▶) and *modiCIFer* (Guzei, 2007 ▶).

## Supplementary Material

Crystal structure: contains datablocks global, I. DOI: 10.1107/S1600536810020143/rk2206sup1.cif

Structure factors: contains datablocks I. DOI: 10.1107/S1600536810020143/rk2206Isup2.hkl

Additional supplementary materials:  crystallographic information; 3D view; checkCIF report

**Fig. 1.**
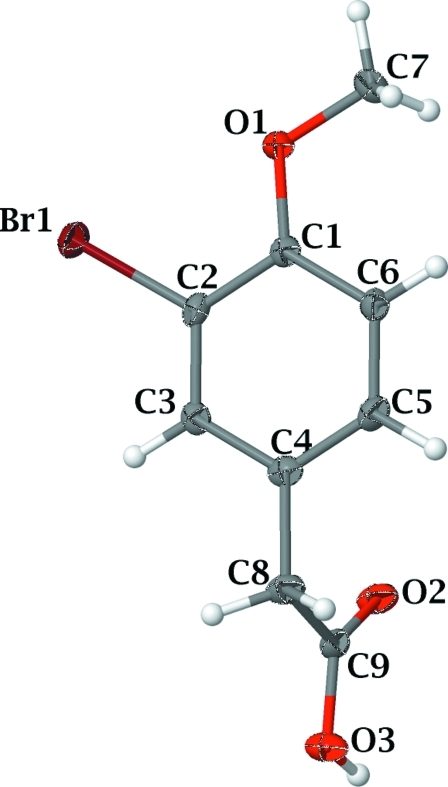
Molecular structure of I with the atom numbering scheme. The displacement ellipsoids are shown at 50% probability level.

Enhanced figure: interactive version of Fig. 1

## Figures and Tables

**Table 1:** Hydrogen-bond geometry (Å, °)

*D*—H⋯*A*	*D*—H	H⋯*A*	*D*⋯*A*	*D*—H⋯*A*
O3—H3⋯O2^i^	0.84	1.82	2.661 (2)	179

Symmetry code: (i) 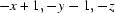.

## supplementary crystallographic information

### Comment

Recently, we have been pursuing simple organic and organometallic compounds as candidates for the introduction of (a) natural product synthesis into the undergraduate teaching laboratory and (b) crystal growing techniques and single crystal X-ray diffraction data analysis into the undergraduate curriculum (Findlater *et al.*, 2010; Guzei *at al.*, 2010a). The 3-bromo-4-methoxyphenylacetic acid I has been employed in the synthesis of natural products such as *Combretastatin A*-4, (Zou *et al.*, 2008), *Verongamine* (Wasserman & Wang, 1998) and model *Vancomycin*-type systems (Ghosh *et al.*, 2009). The iodo-analogue features in the synthesis of the perylenequinones (+)-*Phleichrome* and (+)-*Calphostin D,* (Morgan *et al.*, 2010). Our interest in I stems from its role in the synthesis of the antimitotic compound *Combretastatin A*-4 *via* a simple Perkin condensation/decarboxylation sequence (Zou *et al.*, 2008). This concise route, employing commercially available starting materials, followed by the facile purification of I to furnish high quality crystals makes it ideal in both regards. Compound I is readily synthesized by the regioselective bromination of 4-methoxyphenylacetic acid using bromine in acetic acid (Coutts *et al.*, 1970; Morgan *et al.*, 2007; Zou *et al.*, 2008; Ghosh *et al.*, 2009). Compound I was isolated and characterized by NMR, mp, and single-crystal X-ray analysis. There are three main structural aspects students should identify. First, the positions of the alkyl substituents on the phenyl ring. The methoxy-group is almost coplanar with the ring, torsion angle C7—O1—C1—C6 is 1.2 (3)°, whereas the acetic acid terminus is nearly perpendicular to the ring with the dihedral angle between the planes defined by atoms C1—C6 and atoms C4,C8,C9,O2,O3 spanning 78.15 (7)°. Secondly, the distortions of the C—C—C angles from 120° at the substituents of the phenyl ring reflect their electronic properties. The stronger the electron-withdrawing power of a substituent, the larger the C—C—C angle. The angles at O*Me*, *Ac* and Br are 118.2 (2), 118.4 (2), and 121.5 (2)°, respectively, indicating that the Br atom is electron-withdrawing, whereas the other substituents possess electron-donating properties. Of course, the magnitude of the values is affected by the neighbouring substituents. Thirdly, the molecules of I form centrosymmetric strongly hydrogen-bonded dimers in the lattice. The hydrogen bonding motif is *R*_2_^2^(8). A topical discussion of hydrogen bonding motif assignment was published by Guzei *et al.*, 2010b.

### Experimental

To a stirred solution of 4-methoxyphenylacetic acid (10 g, 60.2 mmol) in acetic acid (60 ml) was added a solution of bromine (9.62 g, 3.1 ml, 60.2 mmol) in acetic acid (30 ml) slowly dropwise over 30 min. The mixture was stirred at room temperature (60 min) and then poured into 500 ml ice–water. The resultant pale yellow, turbid mixture was stirred (10 min), filtered, rinsed with ice–water (3×10 ml), air-dried (20 min) and recrystallized from hot xylene to give a white crystalline powder. Yield 12.41 g, 84 %. M.p. 386.3–387.2 K.

^1^H NMR (CDCl_3_): δ 3.56 (2*H*, s, CH_2_); 3.89 (3*H*, s, OCH_3_), 6.86 (1*H*, d), 7.19 (1*H*, dd), 7.48 (1*H*, d). ^13^C(^1^H) NMR (CDCl_3_): δ 39.9; 56.5; 111.9; 112.2; 127.0; 129.6; 134.6; 155.5; 178.0.

### Refinement

All H-atoms were placed in idealized locations. The C—H distances were 0.98Å for the methyl group, 0.99Å the methylene group, 0.95Å for the *sp*^2^-hybridized atoms; the O—H distance was fixed at 0.84Å. All H atoms were refined as riding with thermal displacement coefficients *U*_iso_(H) set to 1.5*U*_eq_(C, O) for the methyl- and hydroxyl-groups and to to 1.2*U*_eq_(C) for the CH- and CH_2_-groups.

The outlier reflections were omitted based on the statistics test described by Prince & Nicholson, (1983) and Rollett, (1988), and implemented in program FCF_filter (Guzei, 2007). The number of omitted outliers is 4.

### Crystal data

**Table:**

C_9_H_9_BrO_3_	*F*(000) = 488
*M**_r_* = 245.06	*D*_x_ = 1.749 Mg m^−^^3^
Monoclinic, *P*2_1_/*c*	Melting point = 386.3–387.2 K
Hall symbol: -P 2ybc	Cu *K*α radiation, λ = 1.54178 Å
*a* = 12.5022 (4) Å	Cell parameters from 9563 reflections
*b* = 8.2690 (2) Å	θ = 3.5–69.5°
*c* = 9.0199 (3) Å	µ = 5.81 mm^−^^1^
β = 93.573 (1)°	*T* = 120 K
*V* = 930.67 (5) Å^3^	Block, colourless
*Z* = 4	0.46 × 0.37 × 0.19 mm

### Data collection

**Table:**

Bruker SMART APEXII area-detector diffractometer	1725 independent reflections
Radiation source: fine-focus sealed tube	1708 reflections with *I* > 2σ(*I*)
graphite	*R*_int_ = 0.030
0.5° ω and 0.5° φ scans	θ_max_ = 69.5°, θ_min_ = 3.5°
Absorption correction: analytical (*SADABS*; Bruker, 2007)	*h* = −14→15
*T*_min_ = 0.177, *T*_max_ = 0.398	*k* = −9→10
12598 measured reflections	*l* = −10→10

### Refinement

**Table:**

Refinement on *F*^2^	Primary atom site location: structure-invariant direct methods
Least-squares matrix: full	Secondary atom site location: difference Fourier map
*R*[*F*^2^ > 2σ(*F*^2^)] = 0.026	Hydrogen site location: inferred from neighbouring sites
*wR*(*F*^2^) = 0.073	H-atom parameters constrained
*S* = 1.08	*w* = 1/[σ^2^(*F*_o_^2^) + (0.0442*P*)^2^ + 1.1574*P*] where *P* = (*F*_o_^2^ + 2*F*_c_^2^)/3
1725 reflections	(Δ/σ)_max_ < 0.001
120 parameters	Δρ_max_ = 0.86 e Å^−^^3^
0 restraints	Δρ_min_ = −0.47 e Å^−^^3^

### Special details

**Table:**

Geometry. All s.u.'s (except the s.u. in the dihedral angle between two l.s. planes) are estimated using the full covariance matrix. The cell s.u.'s are taken into account individually in the estimation of s.u.'s in distances, angles and torsion angles; correlations between s.u.'s in cell parameters are only used when they are defined by crystal symmetry. An approximate (isotropic) treatment of cell s.u.'s is used for estimating s.u.'s involving l.s. planes.
Refinement. Refinement of *F*^2^ against ALL reflections. The weighted *R*-factor w*R* and goodness of fit *S* are based on *F*^2^, conventional *R*-factors *R* are based on *F*, with *F* set to zero for negative *F*^2^. The threshold expression of *F*^2^ > σ(*F*^2^) is used only for calculating *R*-factors(gt) etc. and is not relevant to the choice of reflections for refinement. *R*-factors based on *F*^2^ are statistically about twice as large as those based on *F*, and *R*-factors based on ALL data will be even larger.

### Fractional atomic coordinates and isotropic or equivalent isotropic displacement parameters (Å^2^)

**Table:**

	*x*	*y*	*z*	*U*_iso_*/*U*_eq_	
Br1	0.253622 (18)	0.20274 (3)	0.38506 (3)	0.02281 (12)	
O1	0.05071 (12)	0.0202 (2)	0.32075 (17)	0.0186 (3)	
O2	0.40281 (14)	−0.3902 (2)	0.05910 (19)	0.0234 (4)	
O3	0.50409 (14)	−0.3283 (2)	−0.12886 (19)	0.0229 (4)	
H3	0.5326	−0.4178	−0.1071	0.034*	
C1	0.12214 (17)	−0.0244 (3)	0.2209 (2)	0.0151 (4)	
C2	0.22340 (18)	0.0476 (3)	0.2339 (2)	0.0148 (4)	
C3	0.30189 (17)	0.0087 (3)	0.1381 (2)	0.0160 (4)	
H3A	0.3701	0.0593	0.1493	0.019*	
C4	0.28118 (18)	−0.1041 (3)	0.0255 (2)	0.0172 (5)	
C5	0.1810 (2)	−0.1765 (3)	0.0129 (3)	0.0201 (5)	
H5	0.1661	−0.2542	−0.0631	0.024*	
C6	0.10183 (18)	−0.1383 (3)	0.1087 (3)	0.0181 (5)	
H6	0.0339	−0.1898	0.0977	0.022*	
C7	−0.05187 (18)	−0.0591 (3)	0.3100 (3)	0.0220 (5)	
H7A	−0.0887	−0.0346	0.2135	0.033*	
H7C	−0.0952	−0.0204	0.3896	0.033*	
H7B	−0.0416	−0.1762	0.3195	0.033*	
C8	0.3654 (2)	−0.1464 (3)	−0.0810 (3)	0.0209 (5)	
H8A	0.4175	−0.0564	−0.0831	0.025*	
H8B	0.3306	−0.1576	−0.1821	0.025*	
C9	0.42502 (19)	−0.3007 (3)	−0.0408 (3)	0.0170 (5)	

### Atomic displacement parameters (Å^2^)

**Table:**

	*U*^11^	*U*^22^	*U*^33^	*U*^12^	*U*^13^	*U*^23^
Br1	0.02043 (17)	0.02308 (18)	0.02445 (17)	−0.00116 (9)	−0.00227 (11)	−0.00996 (9)
O1	0.0164 (7)	0.0206 (8)	0.0193 (8)	−0.0018 (6)	0.0047 (6)	−0.0044 (6)
O2	0.0309 (9)	0.0194 (8)	0.0214 (9)	0.0053 (7)	0.0119 (7)	0.0032 (7)
O3	0.0222 (9)	0.0241 (9)	0.0236 (9)	0.0068 (7)	0.0096 (7)	0.0059 (7)
C1	0.0169 (10)	0.0131 (10)	0.0152 (10)	0.0020 (8)	0.0009 (8)	0.0024 (8)
C2	0.0193 (10)	0.0110 (10)	0.0137 (10)	0.0010 (8)	−0.0029 (8)	−0.0009 (8)
C3	0.0163 (10)	0.0137 (10)	0.0180 (10)	0.0004 (8)	0.0005 (8)	0.0035 (8)
C4	0.0210 (11)	0.0152 (11)	0.0157 (11)	0.0040 (9)	0.0041 (8)	0.0040 (8)
C5	0.0252 (12)	0.0181 (11)	0.0169 (11)	0.0004 (9)	0.0013 (9)	−0.0035 (9)
C6	0.0189 (11)	0.0165 (11)	0.0188 (11)	−0.0030 (9)	0.0005 (9)	−0.0020 (9)
C7	0.0160 (11)	0.0241 (12)	0.0262 (12)	−0.0026 (9)	0.0028 (9)	−0.0010 (10)
C8	0.0246 (12)	0.0211 (12)	0.0176 (11)	0.0032 (10)	0.0072 (9)	0.0016 (9)
C9	0.0178 (11)	0.0188 (12)	0.0145 (11)	−0.0014 (8)	0.0019 (9)	−0.0039 (8)

### Geometric parameters (Å, °)

**Table:**

Br1—C2	1.893 (2)	C4—C5	1.386 (3)
O1—C1	1.359 (3)	C4—C8	1.510 (3)
O1—C7	1.438 (3)	C5—C6	1.390 (3)
O2—C9	1.212 (3)	C5—H5	0.9500
O3—C9	1.326 (3)	C6—H6	0.9500
O3—H3	0.8400	C7—H7A	0.9800
C1—C6	1.394 (3)	C7—H7C	0.9800
C1—C2	1.397 (3)	C7—H7B	0.9800
C2—C3	1.385 (3)	C8—C9	1.510 (3)
C3—C4	1.392 (3)	C8—H8A	0.9900
C3—H3A	0.9500	C8—H8B	0.9900
			
C1—O1—C7	116.85 (18)	C5—C6—H6	120.0
C9—O3—H3	109.5	C1—C6—H6	120.0
O1—C1—C6	124.6 (2)	O1—C7—H7A	109.5
O1—C1—C2	117.3 (2)	O1—C7—H7C	109.5
C6—C1—C2	118.2 (2)	H7A—C7—H7C	109.5
C3—C2—C1	121.5 (2)	O1—C7—H7B	109.5
C3—C2—Br1	119.28 (17)	H7A—C7—H7B	109.5
C1—C2—Br1	119.23 (17)	H7C—C7—H7B	109.5
C2—C3—C4	120.3 (2)	C4—C8—C9	113.34 (19)
C2—C3—H3A	119.9	C4—C8—H8A	108.9
C4—C3—H3A	119.9	C9—C8—H8A	108.9
C5—C4—C3	118.4 (2)	C4—C8—H8B	108.9
C5—C4—C8	120.7 (2)	C9—C8—H8B	108.9
C3—C4—C8	120.9 (2)	H8A—C8—H8B	107.7
C4—C5—C6	121.7 (2)	O2—C9—O3	123.7 (2)
C4—C5—H5	119.2	O2—C9—C8	124.2 (2)
C6—C5—H5	119.2	O3—C9—C8	112.16 (19)
C5—C6—C1	120.0 (2)		
			
C7—O1—C1—C6	1.2 (3)	C3—C4—C5—C6	0.4 (3)
C7—O1—C1—C2	−177.70 (19)	C8—C4—C5—C6	−179.3 (2)
O1—C1—C2—C3	179.39 (19)	C4—C5—C6—C1	0.1 (4)
C6—C1—C2—C3	0.4 (3)	O1—C1—C6—C5	−179.4 (2)
O1—C1—C2—Br1	−1.0 (3)	C2—C1—C6—C5	−0.5 (3)
C6—C1—C2—Br1	180.00 (17)	C5—C4—C8—C9	−81.5 (3)
C1—C2—C3—C4	0.1 (3)	C3—C4—C8—C9	98.8 (3)
Br1—C2—C3—C4	−179.53 (17)	C4—C8—C9—O2	5.8 (3)
C2—C3—C4—C5	−0.5 (3)	C4—C8—C9—O3	−175.0 (2)
C2—C3—C4—C8	179.2 (2)		

### Hydrogen-bond geometry (Å, °)

**Table:**

*D*—H···*A*	*D*—H	H···*A*	*D*···*A*	*D*—H···*A*
O3—H3···O2^i^	0.84	1.82	2.661 (2)	179

Symmetry codes: (i) −*x*+1, −*y*−1, −*z*.
